# Unlocking bimetallic active sites via a desalination strategy for photocatalytic reduction of atmospheric carbon dioxide

**DOI:** 10.1038/s41467-022-29671-0

**Published:** 2022-04-20

**Authors:** Xuezhen Feng, Renji Zheng, Caiyan Gao, Wenfei Wei, Jiangguli Peng, Ranhao Wang, Songhe Yang, Wensong Zou, Xiaoyong Wu, Yongfei Ji, Hong Chen

**Affiliations:** 1 State Environmental Protection Key Laboratory of Integrated Surface Water Groundwater Pollution Control, Guangdong Provincial Key Laboratory of Soil and Groundwater Pollution Control, Key Laboratory of Municipal Solid Waste Recycling Technology and Management of Shenzhen City, Shenzhen Key Laboratory of Interfacial Science and Engineering of Materials (SKLISEM), School of Environmental Science and Engineering, Southern University of Science and Technology, Shenzhen, 518055 China; 2 Hubei Key Laboratory of Mineral Resources Processing and Environment, School of Resources and Environmental Engineering, Wuhan University of Technology, Wuhan, 430070 China; 3 School of Chemistry and Chemical Engineering, Guangzhou University, Guangzhou, 510006 China

**Keywords:** Environmental chemistry, Sustainability

## Abstract

Ultrathin two-dimensional (2D) metal oxyhalides exhibit outstanding photocatalytic properties with unique electronic and interfacial structures. Compared with monometallic oxyhalides, bimetallic oxyhalides are less explored. In this work, we have developed a novel top-down wet-chemistry desalination approach to remove the alkali-halide salt layer within the complicated precursor bulk structural matrix Pb_0.6_Bi_1.4_Cs_0.6_O_2_Cl_2_, and successfully fabricate a new 2D ultrathin bimetallic oxyhalide Pb_0.6_Bi_1.4_O_2_Cl_1.4_. The unlocked larger surface area, rich bimetallic active sites, and faster carrier dynamics within Pb_0.6_Bi_1.4_O_2_Cl_1.4_ layers significantly enhance the photocatalytic efficiency for atmospheric CO_2_ reduction. It outperforms the corresponding parental matrix phase and other state-of-the-art bismuth-based monometallic oxyhalides photocatalysts. This work reports a top-down desalination strategy to engineering ultrathin bimetallic 2D material for photocatalytic atmospheric CO_2_ reduction, which sheds light on further constructing other ultrathin 2D catalysts for environmental and energy applications from similar complicate structure matrixes.

## Abstract (web summary)

Ultrathin two-dimensional metal oxyhalides show excellent photocatalytic properties with unique electronic and interfacial structures. Here, the authors develop a top-down desalination strategy to engineer ultrathin bimetallic two-dimensional material for photocatalytic atmospheric carbon dioxide reduction.

## Introduction

Ultrathin two-dimensional nanomaterials (UTNs) with a typical thickness down to a few nanometres have preserved significant advantages for energy catalysis, environmental remediation, and optoelectronic applications^1,2^. Benefited from their large surface area, well-defined interfacial structure, intrinsic quantum-confined electrons, and tunable band structures, UTNs have been recognized as a class of very promising photocatalysts for CO_2_ reduction reaction (CO_2_RR)^3,4^. With this regard, tremendous research works have been devoted to constructing UTNs with well-defined chemical composition and crystal/electronic structures^1,5^. Among all the reported UTNs, metal oxyhalide UTNs are of particular interest for CO_2_RR, benefiting from the heterogeneity of chemical bonding within their crystal structure, as both the covalent metal-oxygen bonding and soft ionic metal-halide bonding are co-existing within the 2D layer. Anisotropic charge distribution between the metal-oxygen layer and metal-halogen slices are retained, resulting in a prefer-oriented internal electric field within the 2D metal-oxyhalide layer^6,7^. Moreover, when the defect engineering approach has been employed to destruct part of the halide or oxygen atoms, a syngenetic effect of the surface exposed unsaturated metal atoms and appropriate internal electric fields are expected to be coupling together, which may contribute to the excellent charge separation property and outstanding catalytic performance in metallic oxyhalide UTNs^8,9^. Among all the reported metal oxyhalides, 2D bismuth oxyhalides are especially interesting^10,11^. With the alternative arrangement of [Bi_m_O_n_]^(3m−2n)+^ layers and halogen layers, a highly dispersive and spatially anisotropic electronic structure is constructed via the anisotropic *p* and *s-p* hybridization between bismuth and halogen atoms^12^. These structure features provide excellent opportunities to tailor the electronic structure through vacancies modulation or heteroatoms doping for advanced photocatalysis^10,13–18^. Reported examples can be found in Co^2+^ doped Bi_3_O_4_Br atomic layers^19^, V^5+^ doped BiOIO_3_^20^, defect-engineered BiOBr atomic layers^21,22^. Furthermore, different synthetic approaches have been devoted to UTNs synthesis, including chemical vapour deposition^23,24^, wet-chemical exfoliation^25,26^, micromechanical cleavage^27,28^, intercalation^29^, acid-assisted etching^30,31^, etc. Interestingly, most of these efforts have been solely devoted to the synthesis of high-quality monometallic oxyhalide UTNs. The bimetallic oxyhalide UTNs with well-defined elemental ratios and dual-metallic active centres are rarely reported^32^.

In this work, noticing the significant lower hydration energy of the ionic bonding between alkaline metal cations and halide anions within the typical framework structure matrix, herein, we have developed a novel top-down desalination strategy to unlock the bimetallic active sites from a complex parental structure matrix, in which the alkaline-halide salt layers and metal oxyhalide layers are arranged alternately. Specifically, we have synthesized an ultrathin bimetallic oxyhalide layered material Pb_0.6_Bi_1.4_O_2_Cl_1.4_ (denoted as PBOC) from its parental structure Pb_0.6_Bi_1.4_Cs_0.6_O_2_Cl_2_ (denoted as PBCOC). We have achieved high CO_2_RR efficiency on directly converting the atmospheric CO_2_ into solar fuels by employing the ultrathin PBOC as the photocatalyst, superior to its bulk parental material PBCOC. The novel top-down desalination strategy developed in this report provides fresh insights into the design of ultrathin 2D materials with well-defined chemical compositions from their corresponding sophisticated host structural matrixes. It paves the way for constructing prosperous ultrathin 2D catalysts for environmental and energy applications.

## Results

### Top-down synthesis of PBOC

**Fig. 1:**
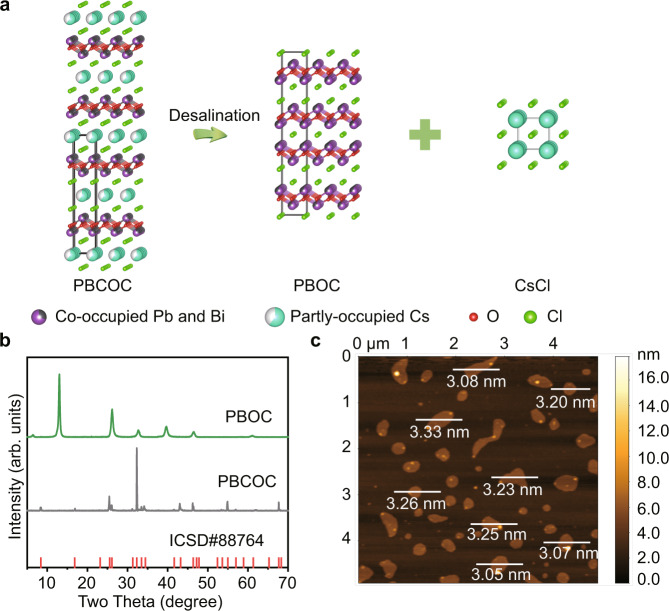
Synthesis and characterization of ultrathin PBOC layers. **a** Schematic illustration of ultrathin PBOC layers synthesis from PBCOC via desalination strategy. **b** PXRD of PBCOC and PBOC. **c** AFM image of the as-synthesized ultrathin PBOC layers.

The desalination strategy developed here is defined as a feasible synthetic strategy to preferably remove the ionically bonded salt-like interlayer within the complicated bulk material, where the anisotropic ionically bonded layer and covalently bonded layer are stacking alternatively. The parental structure of the bulk PBCOC material employed in this study is crystallized in the space group *I*4/mmm (ICSD No.88764)^33^, the partially occupied Cs−Cl layer is alternatively packed within the Pb_0.6_Bi_1.4_O_2_Cl_1.4_ layers as shown in Fig. 1a. The single layer of Pb_0.6_Bi_1.4_O_2_Cl_2_ shows an iso-structure of the tetragonal phase monolayered BiOCl (ICSD No. 74502), in which 30% of the Bi^3+^ positions are randomly occupied by Pb^2+^, while the packing style is significantly different from BiOCl. The interlayered Cs^+^ are bonded with Cl^−^ via ionic bonds, which plays a vital role in neutralizing the extra negative charges within the layer introduced by Pb^2+^ substituting Bi^3+^ (Fig. 1a). Refer to the solubility data in aqueous solution for the corresponding pure binary compounds of all the elements involved in the framework, including CsCl, BiCl_3_, PbCl_2_, Bi_2_O_3_, PbO^34^, Cs−Cl is expected to have a significant water dissolution preference than Bi−Cl, Pb−Cl, Bi−O, and Pb−O bonds within PBCOC. Following the estimated dissolution preference, we anticipate that via a simple ultrasonication assisted process in deionized water, the Cs−Cl layer will be easily dissolved in water thus undergo a desalination process. The PBOC layers in its parental structure of PBCOC will be delaminated to form ultrathin layered materials as schematized in Fig. 1a. With this in mind, a simple ultrasonication process has been employed by loading PBCOC in water. Significant broadening peaks are observed in the powder X-ray diffraction (PXRD) pattern as shown in Fig. 1b for the obtained layered PBOC materials, indicating the layer thickness decreases compared with the original PBCOC bulk material^35^. The diffraction pattern evolution suggests that a crystallographic structure transition has been involved during the desalination process. Atomic force microscopy (AFM) imaging with the corresponding height profiles double confirms that the as-synthesized ultrathin PBOC sheets exhibit an average thickness of 3.2 nm (Fig. 1c and Supplementary Fig. 1). Furthermore, due to the thinner thickness of the obtained material, the Brunauer Emmett Teller (BET) specific surface area (Supplementary Fig. 2) of PBOC increases to 2.561 m^2^ g^−1^, which is 4.3 times larger than that of the original bulk PBCOC (0.5963 m^2^ g^−1^) as measured by N_2_ adsorption and desorption isotherms. Interestingly, when referring to CO_2_ isotherm, PBOC only exhibits a slightly larger CO_2_ BET surface area (2.587 m^2^ g^−1^) than that of PBCOC (1.953 m^2^ g^−1^), as shown in Supplementary Fig. 3, which could be ascribed to the existence of surface Cs^+^ in the parental structure PBCOC. The adsorbed Cs^+^ tend to condense CO_2_ molecules on the bulk material surface thus contributing to a more significant CO_2_ adsorption capacity^36,37^.

**Fig. 2:**
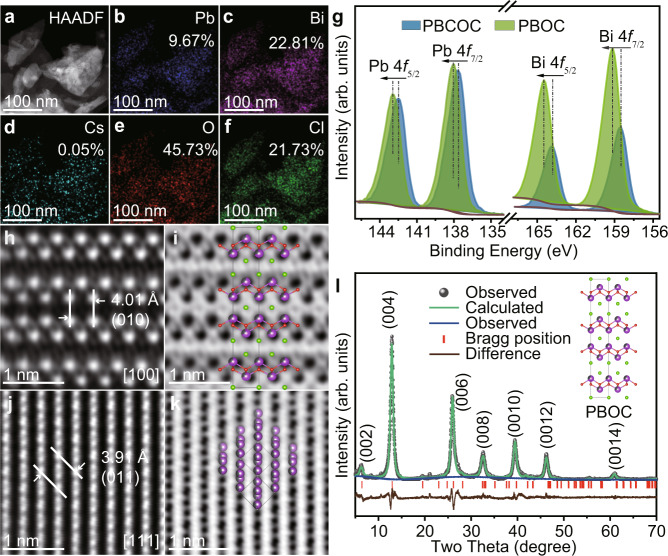
Structure and composition of ultrathin PBOC layers. **a**−**f** STEM-EDS elemental-mapping images and the corresponding element atomic concentrations. **g** Bi 4*f* and Pb 4*f* spectra of ultrathin PBOC layer. **h**−**k** Atom-resolved inversed FFT HAADF- and BF- STEM images, with the labelled zone axis (O and Cl atoms are omitted for the sake of clarity in Fig. **k**). **l** Le Bail fitting results against PXRD pattern.

Scanning electron microscopy (SEM) and scanning transmission electron microscopy (STEM) with its corresponding energy-dispersive X-ray spectroscopy (EDS) mapping (Supplementary Figs. 4 and 5, Fig. 2a−f and Supplementary Table 1) are employed to confirm the morphology and overall elemental distribution in the original bulky PBCOC and the obtained ultrathin PBOC, respectively. Compared with PBCOC, the atomic concentration of Cs in PBOC is lowered down to 0.05%; carefully check the STEM-EDS spectra of PBOC as shown in Supplementary Fig. 6, the characteristic Cs L lines are fully immersed within the background noise, which suggests that the Cs^+^ concentration of 0.05% indexed in the STEM-EDS mapping can be neglectable. Also, the atomic concentration of Cl has been reduced to 21.73%, suggesting that the Cs^+^ and Cl^−^ do undergo a leaching process during the desalination process. The X-ray photoelectron spectroscopy (XPS), including the survey and high-resolution Bi 4*f*, Pb 4*f*, O 1*s,* and Cl 2*p* spectra, are shown in Fig. 2g and Supplementary Fig. 7. The disappearance of Cs^+^ in PBOC after Cs−Cl desalination can be confirmed by the XPS survey spectrum. The valence states of Bi 4*f*, Pb 4*f*, O 1*s,* and Cl 2*p* remain unchanged^38^, although the binding energies slightly shift to a higher energy level similar to BiOCl^39^. This observation could be ascribed to the nature of the Cs−Cl desalination together with the structural rearrangement of the obtained layered structure. Besides, we applied the inductively coupled plasma–mass spectrometry (ICP-MS) and anion chromatography (AC) to trace the dissolution process of PBCOC. The time-dependent concentrations of Cs^+^ and Cl^−^ in deionized water solution show that almost 100% Cs^+^ and 30% of Cl^−^ are dissolved from PBCOC after 42 h ultrasonic treatment (Supplementary Fig. 8a). Besides, we also used aqua regia to digest the resulting PBOC material. The molar ratio of Cs/Pb/Bi in PBOC has been determined to be 0.02738/5.719/14.00 by ICP-MS (Supplementary Fig. 8b), in which the molar ratio of Pb/Bi is close to the corresponding stoichiometric ratio of 0.6/1.4 in PBOC. The detected very trace amount of Cs^+^ might be accounted for the surface adsorption effects on the resulting ultrathin samples. These observations double confirm that the Cs−Cl has been removed from its precursor parental structure, and the resulting ultrathin layer shows a well-defined chemical formula of Pb_0.6_Bi_1.4_O_2_Cl_1.4_. The crystal structure of the as-synthesized ultrathin PBOC layers has been further analyzed with STEM and Le Bail refinement against PXRD. High-angle annular dark-field (HAADF) and bright-field (BF) STEM images were taken to study the heavy atoms and light atoms arrangements for the as-synthesized PBOC. As shown in Fig. 2h, i, the layered stacking feature and the atomic arrangement can be directly observed from the inversed fast Fourier transform (FFT) of the atom-resolved Z-contrast HAADF-STEM (Fig. 2h) and BF-STEM (Fig. 2i) images as viewed along [100] zone axis. In the PBOC structure, four [Pb_0.6_Bi_1.4_O_2_Cl_1.4_] layers are stacking together via the ABBA fashion governed by a mirror symmetry with the internal two B layers sharing one layer of chlorine atoms. The B layers are stacking with the A layers via Van der Waals interaction. Based on this observation, a hypothetical crystal structure model can be proposed with a tetragonal unit cell of *a* = 4.0 Å and *c* = 27.4 Å. HAADF-STEM (Fig. 2j) and BF-STEM (Fig. 2k) images viewing along [111] axis double verify the lattice parameter for *a* axis is ~4.0 Å. A single-unit-cell-thickness structure model can be well imposed with the STEM observation. Moreover, we further employed the PXRD pattern for Le Bail refinement on the proposed structural model for PBOC. The final refinement has been converged to a structure model with space group *I*4/mmm, *a* = 3.895 Å and *c* = 27.186 Å (Supplementary Tables 2, 3). The deduced lattice parameters from PXRD are slightly smaller than that (4.0 Å and ~27.4 Å) as observed from the atom-resolved HAADF-STEM imaging, which could be ascribed to the boundary expansion effects of the ultrathin layers^40^. As shown in Fig. 2l, the experimental PXRD is consistent with the simulated pattern against the proposed structure mode. Due to the highly anisotropic morphology and preferential stacking of the ultrathin PBOC layers, the PXRD gives rise to a family of (00*l*) peaks ascribing to the ultrathin layered feature, which accounts for a slightly significant refinement convergence agreement factor^41^.

To further understand the driving force of the desalination process in PBCOC, we employed theoretical modelling technique to calculate the dissolution energy of Cs−Cl in PBCOC (reaction (1)). By dividing the overall reaction into two steps, as shown in reactions (2) and (3), the reaction energy of reaction (2) can be calculated with density functional theory (DFT). The reaction energy (3) can be referring to the enthalpies for CsCl (s) dissolution (*∆*_sol_*H*^*θ*^: 17.78 kJ mol^−1^)^42^.

1 $${{{{{{\rm{Pb}}}}}}}_{0.6}{{{{{{\rm{Bi}}}}}}}_{1.4}{{{{{{\rm{Cs}}}}}}}_{0.6}{{{{{{\rm{O}}}}}}}_{2}{{{{{{\rm{Cl}}}}}}}_{2}\to {{{{{{\rm{Pb}}}}}}}_{0.6}{{{{{{\rm{Bi}}}}}}}_{1.4}{{{{{{\rm{O}}}}}}}_{2}{{{{{{\rm{Cl}}}}}}}_{1.4}+0.6\,{{{{{{\rm{Cs}}}}}}}^{+}({{{{{\rm{aq}}}}}})\\ +0.6\,{{{{{{\rm{Cl}}}}}}}^{-}({{{{{\rm{aq}}}}}})$$

2 $${{{{{{\rm{PbBi}}}}}}}_{3}{{{{{{\rm{CsO}}}}}}}_{4}{{{{{{\rm{Cl}}}}}}}_{4}\to {{{{{{\rm{PbBi}}}}}}}_{3}{{{{{{\rm{O}}}}}}}_{4}{{{{{{\rm{Cl}}}}}}}_{3}+{{{{{\rm{CsCl}}}}}}({{{{{\rm{s}}}}}})$$

3 $${{{{{\rm{CsCl}}}}}}\,({{{{{\rm{s}}}}}})\to {{{{{{\rm{Cs}}}}}}}^{+}({{{{{\rm{aq}}}}}})+{{{{{{\rm{Cl}}}}}}}^{-}({{{{{\rm{aq}}}}}})$$

To account for fractional occupancy of Pb and simplify the calculation loading, here PbBi_3_CsO_4_Cl_4_ and PbBi_3_O_4_Cl_3_ were adopted to represent the PBCOC and PBOC. A 2 × 2 supercell was constructed, as shown in Supplementary Fig. 9. We find that up to 47.3 kJ energy is required to split up 1 mol CsCl (s) via the desalination reaction (2), whereas the dissolution of 1 mol CsCl (s) to form Cs^+^ (aq) and Cl^−^ (aq) with a concentration of 1 mol L^−1^ is with uphill energy of 18.3 kJ. Therefore, the total reaction energy to drive the overall reaction is 65.6 kJ mol^−1^. Considering the power density of the applied ultrasonication is 0.1 W cm^−2^, when the time is long enough, the ultrasonic energy is sufficient to promote the desalination and delamination process, despite the uncertainty of the energy conversion efficiency. The above coarse modelling results are consistent with our experimental observations and further verify the feasibility of employing this top-down desalination strategy powdered by ultrasonication to synthesize PBOC from PBCOC.

### Electronic structure and photocatalytic CO_2_ reduction performance

**Fig. 3:**
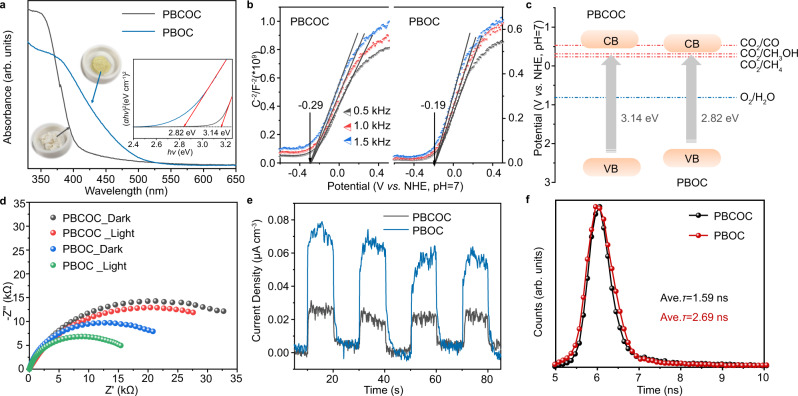
Optical spectroscopy and photoelectrical properties of ultrathin PBOC layers and bulk PBCOC. **a** UV–vis diffuse reflectance spectra. Inset: corresponding optical images and obtained bandgaps of 2.82 and 3.14 eV, estimated by plotting (*αhν*)^2^ versus *hν*. *α* and *ν* are the absorbance and wavenumber; *h* is the Planck constant. **b** Mott−Schottky plots. **c** Schematic illustration of the electronic band structures; grey arrows represent the electron transition process under the light irradiation. CB, conduction band; VB, valence band. **d** Electrochemical impendence spectroscopy. **e** Transient photocurrent densities with light on/off cycles under full spectrum in 0.1 M Na_2_SO_4_ electrolyte solution at an applied potential of 0.5 V vs. Ag/AgCl electrode. **f** Time-resolved fluorescence spectra. Ave. *τ* is the average fluorescence lifetime.

Benefiting from the ultrathin layered feature and rich exposed reconstructed bimetal oxyhalide surface, the ultrathin PBOC exhibits improved electronic structure and photoelectric properties compared with the bulk PBCOC. UV–vis diffuse reflectance spectra displayed in Fig. 3a demonstrates that the bandgap of PBOC is 2.82 eV, which is narrower than its parental structure PBCOC (3.14 eV). Mott–Schottky measurement (Fig. 3b) was conducted to locate the conduction band minimums (CBM) or valence band maximums (VBM) for the ultrathin PBOC and bulk PBCOC. It can be observed that an N-type semiconductor feature is observed in these two materials with their flat-band potentials at −0.19 and −0.29 V, respectively. Accordingly, the experimental CBMs are determined to be at −0.34 and −0.44 V, respectively, since the flat band potential of N-type semiconductors is generally about 0.1 or 0.2 V more positive than its CBMs^43^. The electronic band structures versus NHE at pH 7 can be elucidated in Fig. 3c, which shows that the ultrathin PBOC displays a narrower bandgap than bulk PBCOC due to the potential shifts of both CBM and VBM. This tendency has also been confirmed by DFT calculation (Supplementary Fig. 17a and Fig. 17c). Furthermore, they both show appropriate band edge positions in their electronic structures, which is favourable for the catalytic CO_2_ reduction and O_2_ evolution reactions. Moreover, the electrochemical impendence spectroscopy (EIS) measurements (Fig. 3d) and photoluminescence (PL) spectroscopy results (Supplementary Fig. 10) reveal that PBOC exhibits higher electrical conductivity and lower emission response than PBCOC, especially under light radiation, indicating that PBOC is of improved charge transfer and carriers separation ability. The enhanced transient photocurrent densities in Fig. 3e and time-resolved fluorescence decay spectroscopy in Fig. 3f further confirm the accelerated photoexcited charge carrier transfer dynamics, in which the average lifetime increases from 1.59 ns (PBCOC) to 2.69 ns (PBOC), further suggesting the higher efficiency of charge separation under light irradiation with a slower recombination rate.

**Fig. 4:**
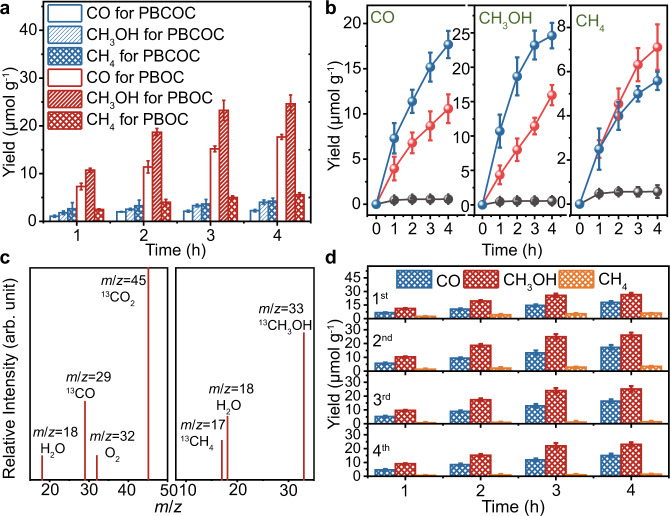
Photocatalytic CO_2_RR performances of the ultrathin PBOC layers and bulk PBCOC. **a** Photocatalytic CO_2_RR products under full-spectrum light irradiation. **b** Photocatalytic CO_2_ reduction activities of the ultrathin PBOC in N_2_ (grey ball), atmospheric CO_2_ (red ball), and pure CO_2_ (blue ball) under full-spectrum light irradiation. **c** GC-MS spectrum of the products with ^13^CO_2_. **d** CO_2_RR cycling measurements under full-spectrum light irradiation for 4 h. Error bars are means ± standard deviation of three replicates.

Inspired by the significantly improved light absorption, charge separation, and transfer abilities of ultrathin PBOC, it is promising to employ PBOC for photocatalytic CO_2_RR (Supplementary Fig. 11). As shown in Fig. 4a, apparent CO_2_ reduction performance, with reaction products of CO, CH_3_OH, and CH_4,_ is achieved with respect to a pure CO_2_ concentration of 1500 ppm under the full spectrum irradiation with a standard Xe lamp. The dominant evolution products for CO and CH_3_OH are approximately 17.91 and 26.53 μmol g^−1^ within 4 h, which are 7.2 and 7.3 times higher than catalyzed by bulk PBCOC. Continuous O_2_ is also produced in the reaction system with a generation rate of ca. 48.69 μmol g^−1^ in 4 h (Supplementary Fig. 12a), which should be the oxidative product of H_2_O. The electrons involved in the reduction reaction are nearly equal to those participating in the oxidation process. Besides, ultrathin PBOC exhibits visible-light-induced photocatalytic CO_2_ reduction property (Supplementary Fig. 12b). Similar CO_2_RR activity was also detected using atmospheric air as a CO_2_ source under the entire solar spectrum, where the CO_2_ concentration in atmospheric air was measured to be 500 ppm with gas chronometry (GC) (Fig. 4b). This observation indicates the existence of oxidative O_2_ in the atmosphere does not influence the CO_2_ reduction performance on PBOC. Besides, as shown in Supplementary Figs. 12c, d, PBOC offers decent photocatalytic CO_2_ reduction activities under different CO_2_ concentrations (500, 1000, and 1500 ppm) and different seasons (Summer: 490−500 ppm CO_2_, 33.0 ± 0.5 °C, 42% humidity; Autumn: 500−510 ppm CO_2_, 22.0 ± 0.5 °C, 60% humidity), in which high temperature is more favourable to the process than high humidity. To confirm the origin of the carbon and oxygen sources in photocatalytic reaction, controlled experiments with ^13^C and ^18^O isotopic tracing were conducted and monitored with a gas chromatography-mass spectrometer (GC-MS) (Fig. 4c and Supplementary Figs. 13, 14). In the ^13^C isotopic tracing experiment, ^13^CO (*m*/*z* = 29), ^13^CH_3_OH (*m*/*z* = 33), ^13^CH_4_ (*m*/*z* = 17) and O_2_ (*m*/*z* = 18) are observed in the GC-MS spectrum; While in ^18^O isotopic tracing experiment, ^18^O_2_ (*m*/*z* = 36) is also detected. These results imply that the above products do convert from CO_2_ reacting with H_2_O. Throughout four cycles (16 h) tests, this ultrathin layered catalyst does not exhibit significant catalytic performance decay (Fig. 4d), and structure or composition degradation (Supplementary Fig. 15), which suggests a good photostability. As expected, the PBOC displays outstanding photocatalytic CO_2_ reduction performance over the bulk sample, mainly due to the considerable enhancement of charge separation efficiency, improved light absorption capability, and the declined energy barriers for CO_2_ reduction. Moreover, the photocatalytic CO_2_ reduction properties of PBOC also outperform the as-prepared BiOCl nanosheet (~4.35 nm, Supplementary Fig. 16) and many other state-of-the-art photocatalysts (Supplementary Table 6) under comparable conditions^44^, which could be ascribed to the unique interfacial and electronic structure induced by the mixing occupancy of Pb and Bi in ultrathin PBOC inherited from its parental structure.

### Structural insights of photocatalytic activity

**Fig. 5:**
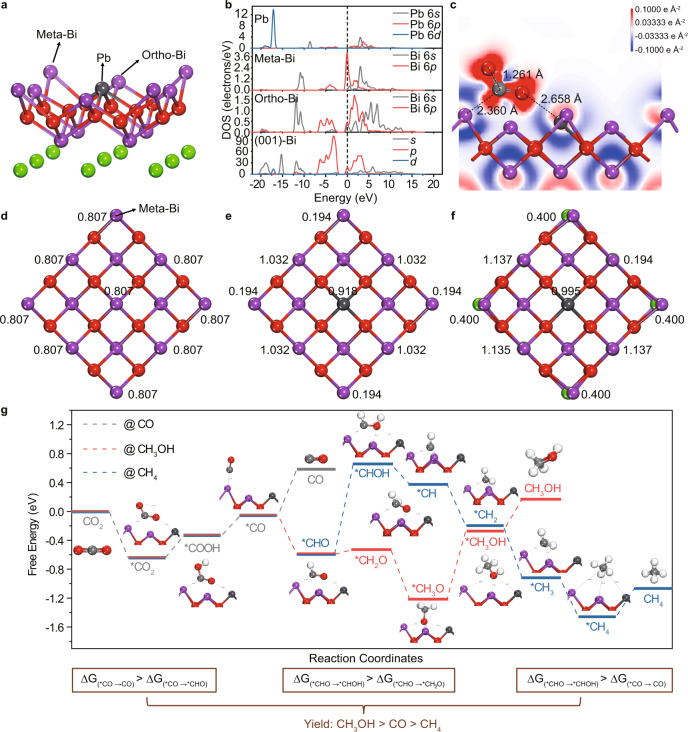
Electronic structure and CO_2_RR pathways of ultrathin PBOC layers. **a** Lead–bismuth oxyhalide outer layer structural model and (**b**) the partial density of states (PDOS). **c** Isosurface of differential charge density. Mulliken population of (**d**) layer bismuth oxyhalide layer, **e** lead–bismuth oxyhalide outer layer and (**f**) lead–bismuth oxyhalide outer layer after CO_2_ adsorption. **g** Gibbs free energy calculations towards the reaction pathway and free energy diagrams. The grey, red and blue lines show the reaction pathway of CO_2_RR to CO, CH_3_OH, and CH_4,_ respectively.

To further understand the intrinsic photocatalytic CO_2_RR reaction dynamics on PBOC interface, DFT calculations were employed to calculate the electronic structure for the outer layer of the single-unit-cell PBOC layer. Interestingly, the co-occupied Pb contributes to a bandgap decrease compared with BiOCl (Supplementary Fig. 17b, c). Besides, the co-occupied Pb can induce a more significant polarization effect, thus promoting photoexcited charge separation and facilitating photocatalytic efficiency^45^. Furthermore, we conducted DFT calculations on a top-site surface model to simulate the charges behaviours and interaction with CO_2_ on PBOC (Supplementary Fig. 18). The model and the partial density of states (PDOS) are shown in Fig. 5a, b suggesting that the co-occupied Pb would induce a higher PDOS on meta-Bi atom at the conduction-band edge. The meta-Bi atom and Pb atom co-exhibit a robust electrostatic attraction towards C and O of the CO_2_ molecule, causing the O=C=O bending as confirmed by the differential charge density map in Fig. 5c. Mulliken population analysis of Pb atom and meta-Bi atoms (Fig. 5d, e) indicate that Pb can significantly suppress the electron donor of meta-Bi, resulting in a large decrease of Mulliken charge from 0.807 to 0.194. The meta-Bi atoms with affluent electron density exhibit a strong electrostatic attraction towards electrophilic carbon atoms in CO_2_ molecules (Fig. 5c). After binding with CO_2_, the high electron intensity around meta-Bi atom is neutralized, with the Mulliken charge increasing from 0.194 to 0.400 (Fig. 5f), revealing that electron transfer occurred between meta-Bi atom and CO_2_. The Pb atom plays a vital role in PBOC for the photogenerated charge separation and transfer in the photocatalytic CO_2_RR process, in which the CO_2_ molecules can be sufficiently adsorbed and activated (CO_2_ → *CO_2_), thus facilitating the subsequent reduction reactions.

Based on the CO_2_ molecules activation process^46,47^, we further explore the CO_2_RR dynamics on the surface of ultrathin PBOC. Three typical possible reaction pathways of reducing CO_2_ to CO, CH_3_OH, and CH_4_ were considered (Supplementary Note 1, Supplementary Tables 4, 5, and Fig. 5g), respectively. According to the detailed reaction pathways with the diagram illustrated in Fig. 5g, the CO_2_ on the ultrathin PBOC interface is initially activated to generate the adsorbed *CO_2_, which is subsequently energetically uphill transformed to *COOH and *CO. Afterwards, the transition from *CO to *CHO is thermodynamically favourable with an energy release of 0.651 eV, while the desorption of CO requires an energy of 0.262 eV. Thus, *CO is more feasible to be transformed into *CHO following pathway II or III. This explains the higher productivity of CH_3_OH and CH_4_ on ultrathin PBOC layers in CO_2_RR. Then, the reduction energy barrier from *CHO to *CHOH (pathway III) is much more significant than that to form *CH_2_O (pathway II), which is consistent with the observation of lower CH_4_ yield during the CO_2_RR process. Overall, the energy expenditure from *CHO to *CHOH (pathway III) is higher than that from *CO to CO (pathway I), further verifying the CO_2_RR yield ranking of CH_3_OH > CO > CH_4_, which is consistent with the experimental observations.

## Discussion

Via a top-down desalination strategy, we have successfully unlocked the rich active bimetallic interface from its parental structure PBCOC. The as-synthesized ultrathin PBOC material exhibits a larger surface area with fruitful well-defined bimetallic catalytic centres, which enhanced the photocatalytic CO_2_RR performance in gas-phase reaction, with the atmospheric CO_2_ as gas source. Based on the insightful mechanistic study on the structural-property relationship, the unlocked rich catalytic sites, enhanced interlayer charge conductivity, and superior structural stability are critical factors contributing to the excellent CO_2_RR performance. The novel desalination strategy used to unlock the active intralayer interface and reactive centres, can be extended to fabricating other UTNs from their complicated parental structures that with covalent and ionic bonded layers co-existed. This work not only reports a novel, highly efficient ultrathin photocatalyst for atmospheric CO_2_ reduction and paves the way for the carbon-neutral global, but it also expands the avenue for designing and synthesizing other ultrathin 2D materials with complex compositions from the well-documented host structural matrixes for various applications.

## Methods

### Material synthesis

All the chemical reagents, including bismuth nitrate pentahydrate (Aladdin, ACS, ≥98.0%), sodium chloride (Aladdin, AR, 99.5%), caesium chloride (Alfa Aesar, metals basis, 99.999%), lead oxide yellow (Aladdin, AR, 99.9%), and NaOH (Innochem, 99%), were used as purchased without further purification. The layered ultrathin BiOCl was synthesized via a hydrothermal route^7^. 1 mmol of Bi(NO_3_)_3_ ∙ 5H_2_O and 1 mmol of KCl were added in 15 mL distilled water with continuous stirring, and then 1 M NaOH solution was used to adjust the pH to neutral. After stirring for 30 min, the mixture was transferred to a 20 mL Teflon-lined stainless autoclave and heated at 160 °C for 24 h, then air-cooled to room temperature. PBCOC was synthesized via a solid-state chemical reaction under vacuum. 1.4 mmol (0.3647 g) BiOCl, 0.6 mmol (0.1339 g) PbO, and 0.6 mmol (0.1015 g) CsCl were weighted in an Ar-filled glovebox and grounded in a mortar for 30 mins. The well-grounded precursor powder was sealed in a 9 mm-diameter quartz ampoule. Then the ampoule was loaded into a programmable muffle furnace and heated to 800 °C at a rate of 10 °C min^−1^. The ampoule was maintained in the furnace at 800 °C for 5 days and naturally cooled down to room temperature. White PBCOC powders have been harvested. Afterwards, 1.0000 g of PBCOC was loaded in a 250 mL Erlenmeyer flask and mixed with 150 mL of deionized water. The mixture was periodically treated with an ultrasonic bath (80 Hz, 100 W) for 14 days (ultrasonicated for 3 h and swell on standing for 9 h, twice every day), ultrathin PBOC layers were harvested after filtering the resulted solution.

### Characterization

PXRD patterns were recorded using a 9 KW Rigaku SmartLab diffractometer with Cu K_α_ radiation (λ = 1.5406 Å). The thickness measurement of the samples was performed on AC Mode AFM (Asylum Research, MFP-3D-Stand Alone). BET-specific surface areas of the as-synthesized materials were determined by N_2_ adsorption/desorption curve on a BELSORP-max machine. The morphology and energy dispersive X-ray spectroscopy study of PBCOC was performed on a Zeiss Merlin SEM operated at various acceleration voltages. The HAADF-STEM imaging and the corresponding EDS analyses of PBOC were performed on an FEI Titan Themis apparatus with an X-FEG electron gun and a DCOR aberration corrector operating at 300 kV. The bulky-like HAADF-STEM image was taken from an isotropic air-dried particle with multiple ultrathin layers aggregated together in the obtained PBOC sample. The XPS was implemented on a PHI 5000 Versaprobe III instrument (ULVAC-PHI, UK) to analyze the valence states of the elements with a monochromatic Al Kα source. The spectrum was analyzed by the PHI-MultiPak software, referencing C1*s* to 284.8 eV. The UV–Vis diffuse reflectance spectra (DRS) of the powder samples were recorded on a UV–Vis-NIR spectrometer (PerkinElmer, Lambda 750S) equipped with an integrating sphere. BaSO_4_ powder was used as a reference. A RF-5301PC PL spectrofluorometer with an excitation wavelength of 200 nm was used to examine the charge recombination rate. The reduction products were analyzed with an Agilent GC-MS. The ions concentrations of PBCOC were determined by ICP-MS (Thermo Fisher iCAP RQ) and Anion Chromatography (Aquion, Thermo Fisher).

### Photocatalytic CO_2_ reduction tests

Photocatalysis experiments were carried out in a custom-made glass vessel with a quartz glass cap (Perfectlight, China, Supplementary Fig. 11)^37^. A 300 W Xe lamp (Perfectlight, China) was used as the full spectrum light source, while the visible light source was obtained by employing a 420 nm filter to exclude the UV light from the full spectrum. The relatively low concentration of CO_2_ (ca.1500 ppm) used in the standard catalysis reaction was in situ generated by the reacting NaHCO_3_ with H_2_SO_4_ (volume ratio of concentrated H_2_SO_4_ and deionized water was 1:1). The in-house atmospheric air with a concentration of ca.500 ppm as characterized with GC was also used as a CO_2_ source for photocatalytic CO_2_RR. The detailed procedures were as follows: firstly, 25 mg of the photocatalyst was uniformly dispersed into 1 mL deionized water and then dried in a vessel at a 60 °C oven. Afterwards, 50 μL of deionized water was dropped onto the catalyst’s surface to construct a humid interface. Next, 500 mg NaHCO_3_ was added to a 200 mL of glass reaction chamber, followed by adding the above-prepared sample vessel on the top of NaHCO_3_ powder in the chamber. Subsequently, the reaction chamber was purged with pure N_2_ gas for 30 min to expel the air and then vacuumed for 15 min. Before light irradiation, 2 mL of H_2_SO_4_ was injected into the reaction chamber to start producing a low concentration of CO_2_. At a fixed interval period, 200 µL of the gas products were withdrawn and qualitatively analyzed with gas chromatography. The ^13^CO_2_ photoreduction experiment was also carried out in a customized glass vessel with the ^13^CO_2_ source produced by reacting NaH^13^CO_3_ with H_2_SO_4_.

### Le bail refinement

Le Bail refinement was conducted to confirm the lattice parameters and crystal structure of PBOC using the GSAS-EXPGUI suite^48^. Here, the primary structural model of PBOC with a space group of *I*4/mmm was proposed from the atom-resolution HAADF-STEM imaging.

### Solvation calculation

First-principles calculation was performed with DFT implanted in VASP at spin-polarized generalized gradient approximation (GGA) level^49^. PBE exchange-correlation functional and PAW pseudo potential were used. An energy cut-off of 520 eV was applied for the plane-wave basis set. The Brillouin zones were sampled by a 3 × 3 × 1 grid of Monkhorst-pack k points. All atoms were allowed to relax until the maxima force on the atoms was smaller than 0.02 eV Å^−1^.

### DFT calculations

DFT calculation was performed using DMol^3^ code^50,51^. The Perdew-Burke-Ernzerhof (PBE) exchange-correlation functional with GGA was utilized to describe the exchange-correlation energy^52,53^. Spin-polarization was included in all calculations, and a damped van der Waals correction was incorporated using Grimme’s scheme to describe the non-bonding interactions^54^. The density functional semi core pseudo potential (DSPP) was utilized to account for the relativistic effects of core electrons and the double numerical plus polarization (DNP) basis set^55,56^. The *k*-point of 4 × 4 × 2 and 2 × 2 × 1 were set for the bulk and slab model using the Monkhorst-Pack method, respectively.

To better evaluate the specific active catalytic site within PBOC, Bi-terminated Pb_0.6_Bi_1.4_O_2_Cl_1.4_ (001) in a 2 × 2 × 1 supercell was constructed to represent the out layer of PBOC, in which a top-site lead-bismuth oxyhalide layer with Pb proportion of 25% was modulated to replace the Pb/Bi mixing occupancy surface with Pb proportion of 30%, as illustrated in Supplementary Fig. 18.

To better simulate the experimental environment, a water solvation model (COSMO) with a dielectric constant of 78.54 was applied to mimic the aqueous condition. The change in Gibbs free energy (*ΔG*) for each of the CO_2_RR steps was evaluated according to the computational hydrogen electrode (CHE) model suggested by Nørskov and co-workers^57,58^. The Gibbs free energy was defined as:

4 $$\varDelta G=\varDelta {E}_{{{{{\rm{DFT}}}}}}+\varDelta {E}_{{{{{\rm{ZPE}}}}}}{-}T\varDelta S$$

where *ΔE*_DFT_ is the electronic energy difference directly obtained from DFT calculation, *ΔE*_ZPE_ and *TΔS* are the zero-point energy correction and entropy change at *T* = 298.15 K, respectively. Here, the correction terms are present in Supplementary Table 4. Most of the corrections are from previous literature^58,59^. In addition, −0.41 eV correction was applied to the electronic energy of CO as the PBE functional cannot describe this molecule accurately^58,60^.

## Supplementary information

Supplementary Information

## Data Availability

All the data supporting the findings of this study are available within the paper and its supplementary information files.
