# Supplementary material for: Unlocking bimetallic active sites via a desalination strategy for photocatalytic reduction of atmospheric carbon dioxide
Source: Nat Commun. 2022 Apr 20;13:2146. doi: 10.1038/s41467-022-29671-0 (PMC9021305; doi:10.1038/s41467-022-29671-0)
Supplement: Supplementary file 1 — Supplementary Information [file 41467_2022_29671_MOESM1_ESM.pdf]

Supplementary Information for

Unlocking Bimetallic Active Sites via a Desalination Strategy for Photocatalytic Reduction of Atmospheric Carbon Dioxide

Xuezhen Feng[1,†], Renji Zheng[1,†], Caiyan Gao[2], Wenfei Wei[1], Jiangguli Peng[1], Ranhao Wang[1], Songhe Yang[1], Wensong Zou[1], Xiaoyong Wu[2,*], Yongfei Ji[3], Hong Chen[1,*]

[1]State Environmental Protection Key Laboratory of Integrated Surface Water Groundwater Pollution Control, Guangdong Provincial Key Laboratory of Soil and Groundwater Pollution Control, Key Laboratory of Municipal Solid Waste Recycling Technology and Management of Shenzhen City, Shenzhen Key Laboratory of Interfacial Science and Engineering of Materials (SKLISEM), School of Environmental Science and Engineering, Southern University of Science and Technology, Shenzhen 518055, China.

[2]Hubei Key Laboratory of Mineral Resources Processing and Environment, School of Resources and Environmental Engineering, Wuhan University of Technology, Wuhan 430070, China.

[3]School of Chemistry and Chemical Engineering, Guangzhou University, Guangzhou 510006, China.

[†]These authors contributed equally: Xuezhen Feng, Renji Zheng

*These authors jointly supervised this work: Xiaoyong Wu (parawu521@163.com); Hong Chen (chenh3@sustech.edu.cn)

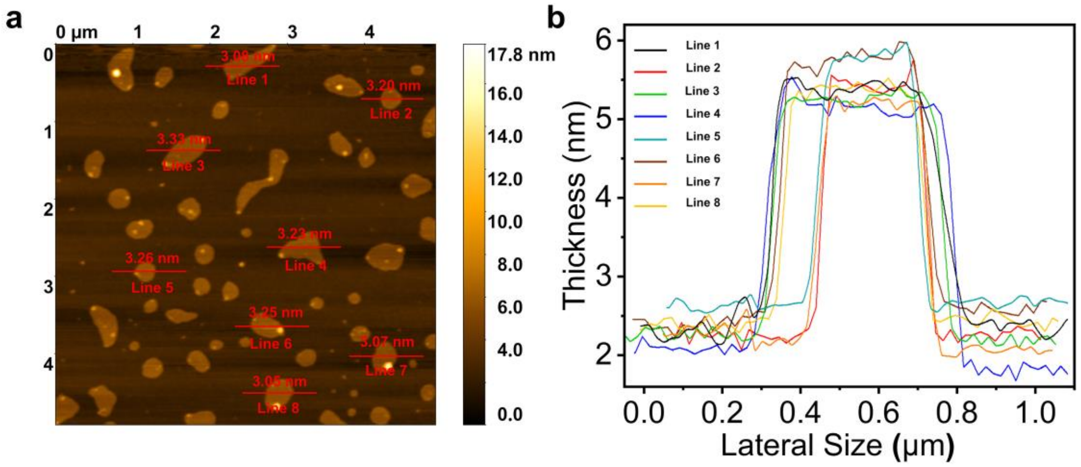

Supplementary Fig. 1 AFM analysis. a AFM image and **b** the corresponding height profiles of PBOC.

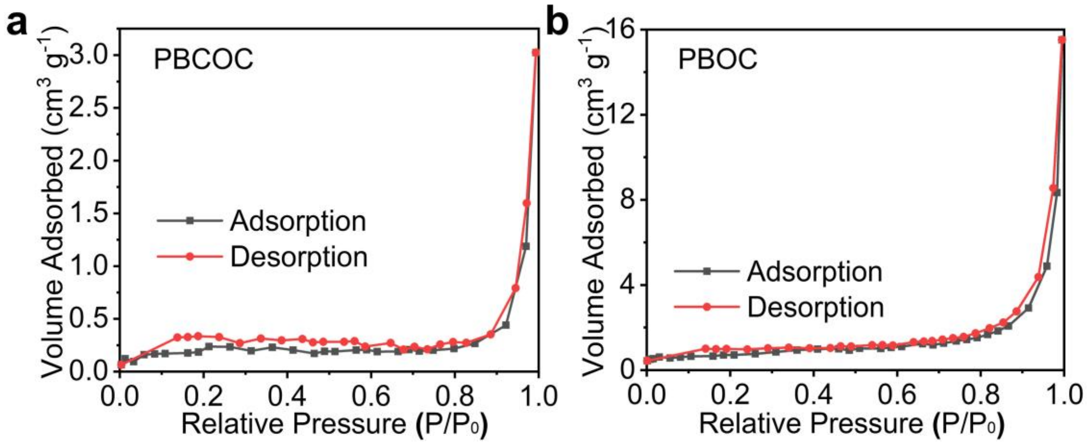

Supplementary Fig. 2 N_2 adsorption and desorption isotherms. N_2 BET surface area of **a** PBCOC: 0.5963 m^2 g^{-1}. **b** PBOC: 2.561 m^2 g^{-1}

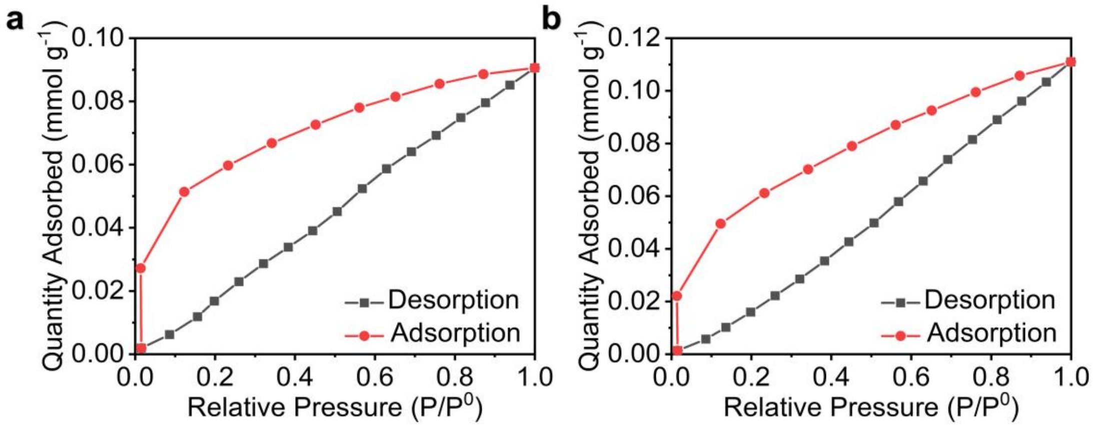

Supplementary Fig. 3 CO_2 adsorption and desorption isotherms. CO_2 BET surface area of **a** PBCOC: 1.953 m^2 g^{-1}. **b** PBOC: 2.587 m^2 g^{-1}.

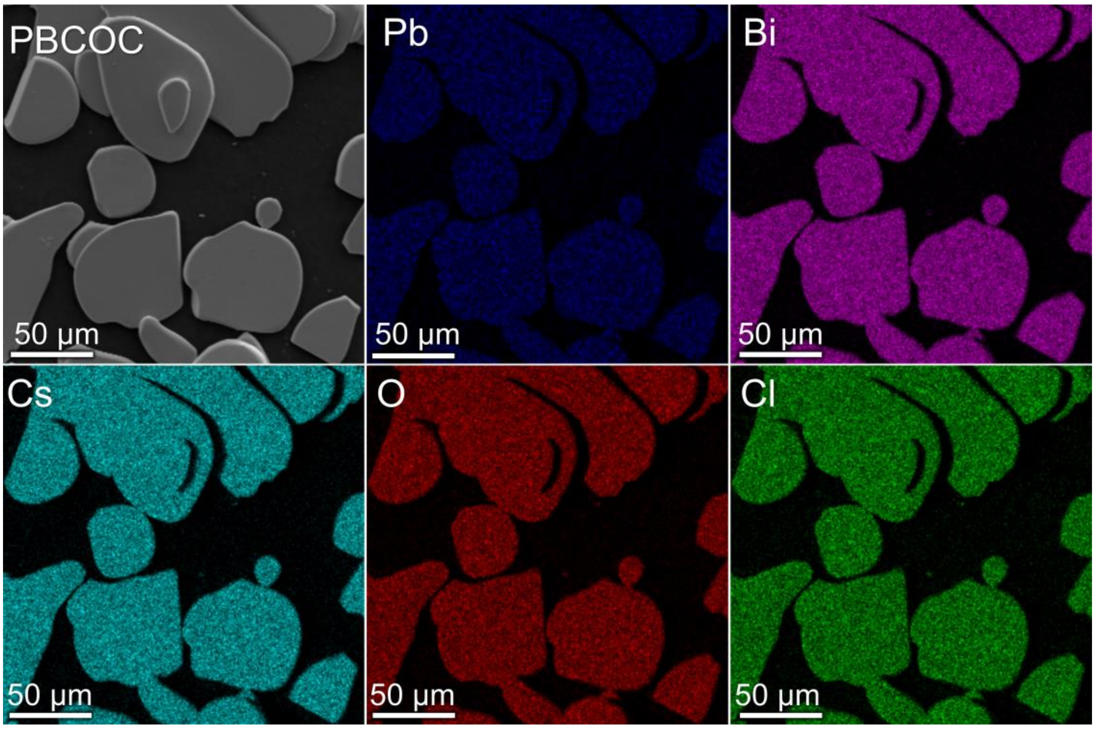

Supplementary Fig. 4 SEM Morphology and EDS elemental mapping images of PBCOC.

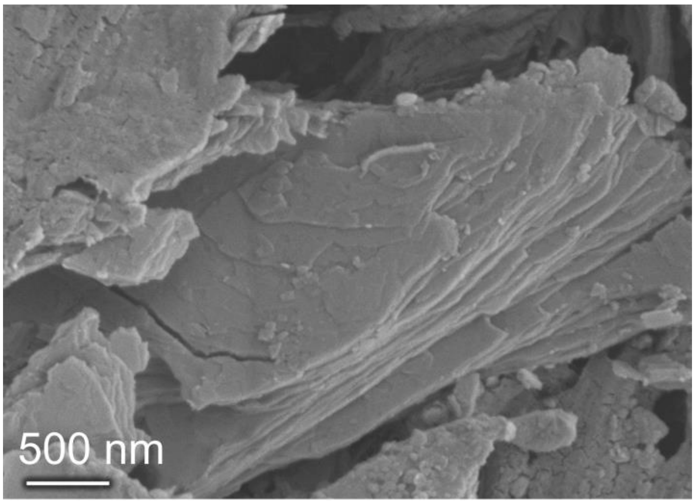

Supplementary Fig. 5 SEM Morphology of PBOC.

Supplementary Table 1 Element atomic concentrations derived from EDS for bulk PBCOC, PBOC and their corresponding theoretical stoichiometric values.

Element Atomic Concentrations (%)		Pb M	Bi M	Cs L	O K	Cl K
PBCOC	SEM-EDS	8.280	16.10	9.670	32.72	33.32
	Stoichiometric	9.090	21.21	9.090	30.30	30.30
PBOC	STEM-EDS	9.670	22.81	0.050	45.73	21.73
	Stoichiometric	11.11	25.93	0.000	37.04	25.93

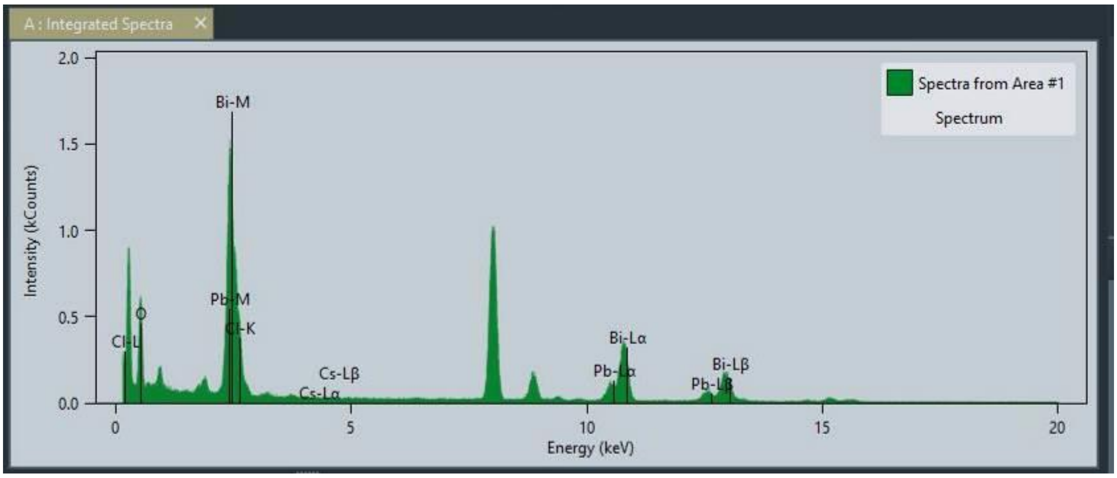

Supplementary Fig. 6 Integrated spectrum of STEM-EDS mapping for PBOC.

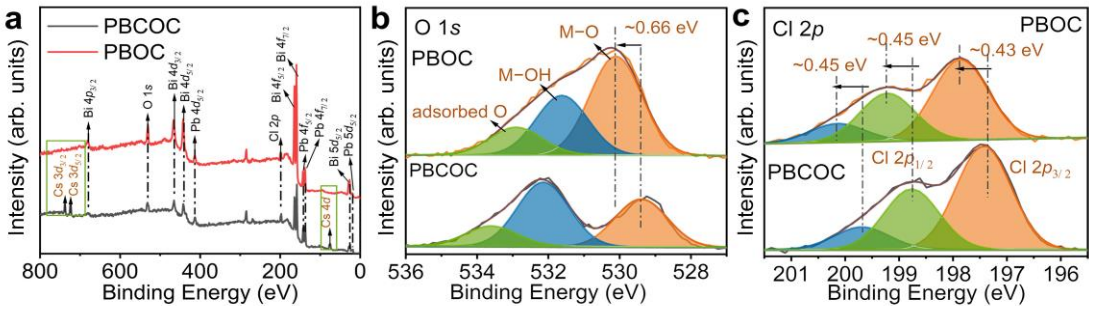

Supplementary Fig. 7 XPS spectra of bulk PBCOC and PBOC. a Survey spectra. **b** O 1*s* and **c** Cl 2*p*.

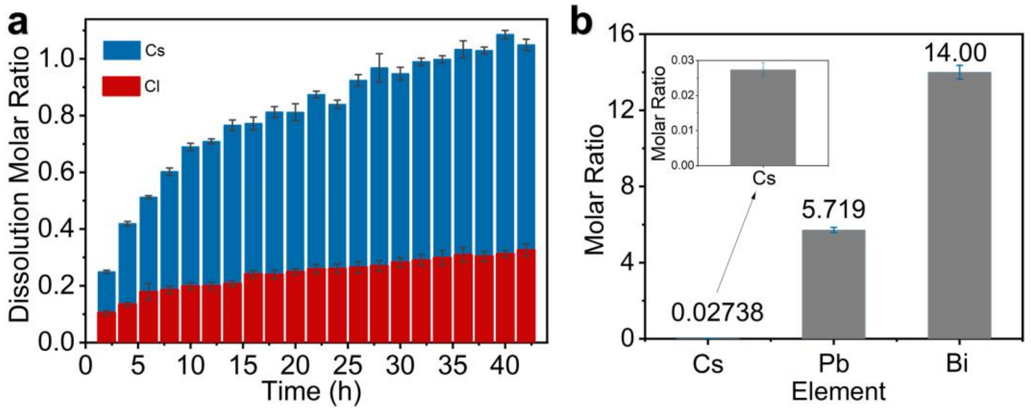

Supplementary Fig. 8 Time-dependent composition evolution during the desalination process. a molar ratio of Cs^+ and Cl^- in deionized water as dissolved from original PBCOC under different ultrasonication time. **b** Cs/Pb/Bi molar ratio within the obtained ultrathin PBOC layers. Error bars are means ± standard deviation of three replicates.

Supplementary Table 2 Le Bail refinement results of PBOC against PXRD.

Compound	Space Group	*a* (Å)	*c* (Å)	V_{cell} (Å^3)	χ^2	R_p	R_{wp}
PBOC	*I*4/mmm	3.895 (1)	27.19 (2)	412.5 (1)	4.158	0.1090	0.1540

Supplementary Table 3 Atomic positions of proposed PBOC structure from Le Bail refinement.

Atom	*X*	*Y*	*Z*	Occupancy Ratio
Pb1	0.5000000(0)	-0.5000000(0)	0.0688500(0)	0.3
Bi1	0.5000000(0)	-0.5000000(0)	0.0688500(0)	0.7
Cl2	0.5000000(0)	-0.5000000(0)	-0.2074600(0)	1
Pb3	0.5000000(0)	-0.5000000(0)	0.6609600(0)	0.3
Bi3	0.5000000(0)	-0.5000000(0)	0.6609600(0)	0.7
Cl4	0.5000000(0)	-0.5000000(0)	0.5000000(0)	1
O5	0.5000000(0)	0.0000000(0)	-0.1115100(0)	1

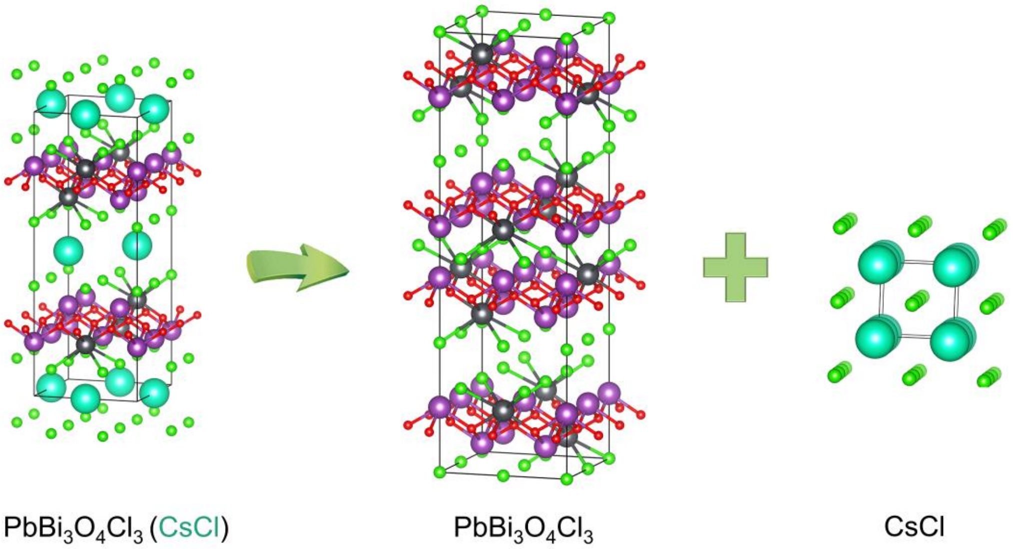

Supplementary Fig. 9 Structures of $PbBi_3CsO_4Cl_4$ and $PbBi_3O_4Cl_3$ used for modeling.

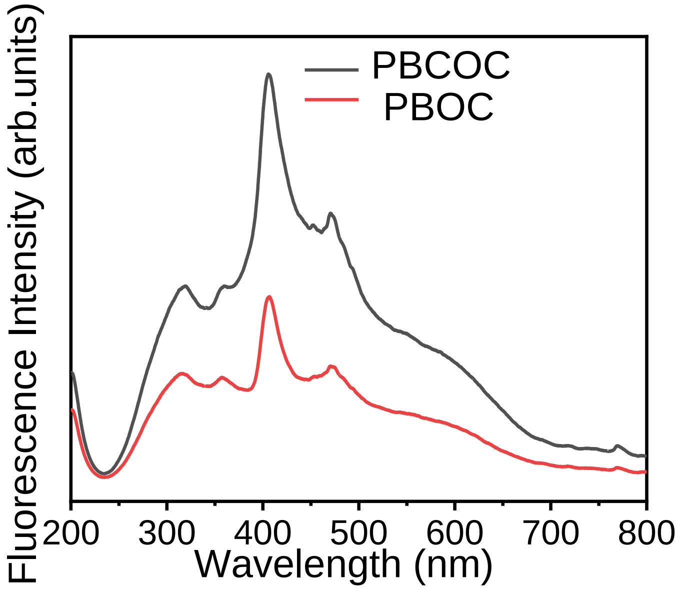

Supplementary Fig. 10 Photoluminescence spectroscopy of the bulk PBCOC and the ultrathin PBOC. Note: the excitation wavelength is 200 nm, the sharp peak at 400 nm is originated from the frequency-doubled reflection peak.

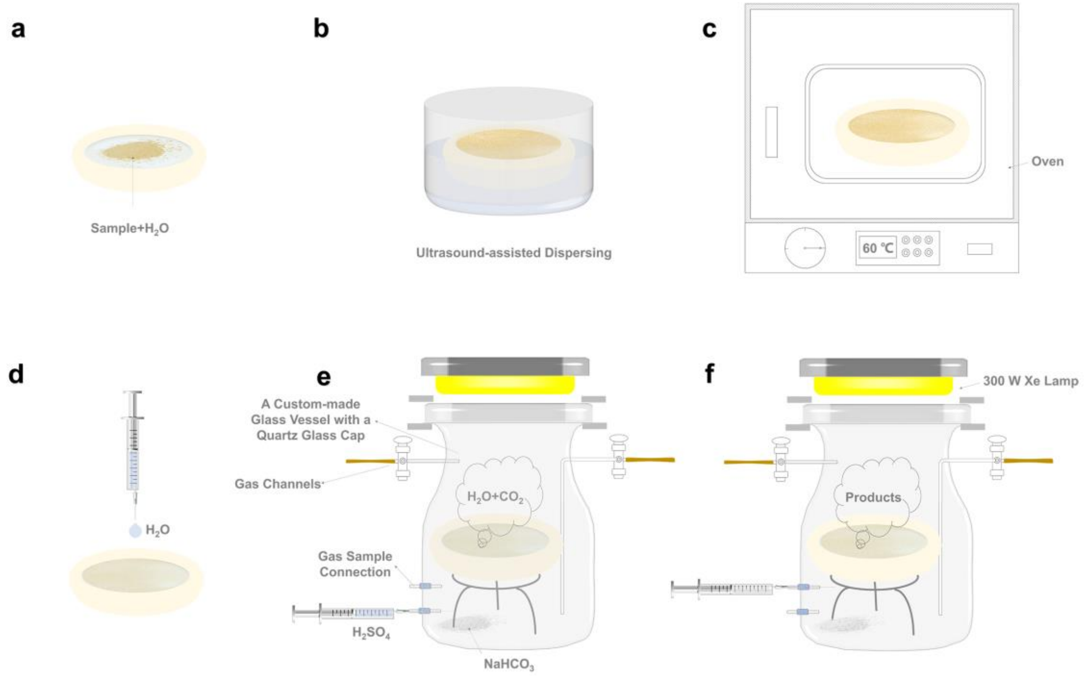

Supplementary Fig. 11 Detailed experimental procedure for photocatalytic CO_2RR.

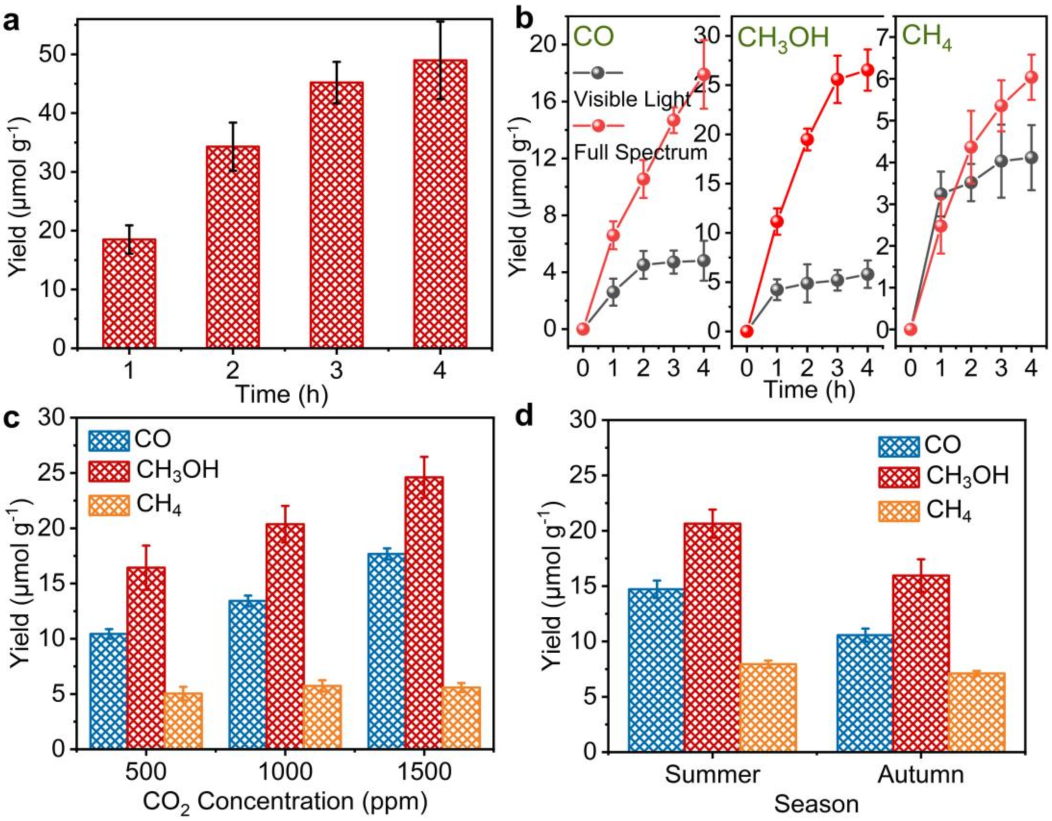

Supplementary Fig. 12 Photocatalytic CO_2RR performances of ultrathin PBOC layers. a O_2 production. **b** Photocatalytic CO_2 reduction activities under different light sources. **c** Photocatalytic CO_2 reduction activities under different CO_2 concentrations. **d** Photocatalytic reduction activities under different seasons with atmospheric air as CO_2 source (Summer: 490−500 ppm CO_2, 33.0 ± 0.5 °C, 42% humidity; Autumn: 500−510 ppm CO_2, 22.0 ± 0.5 °C, 60% humidity). Error bars are means ± standard deviation of three replicates.

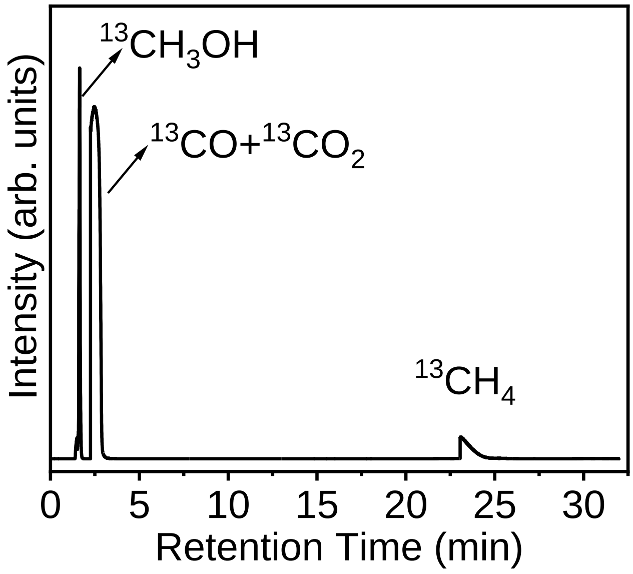

Supplementary Fig. 13 LC-MS spectrum of products from $^{13}CO_2$ photoreduction experiment.

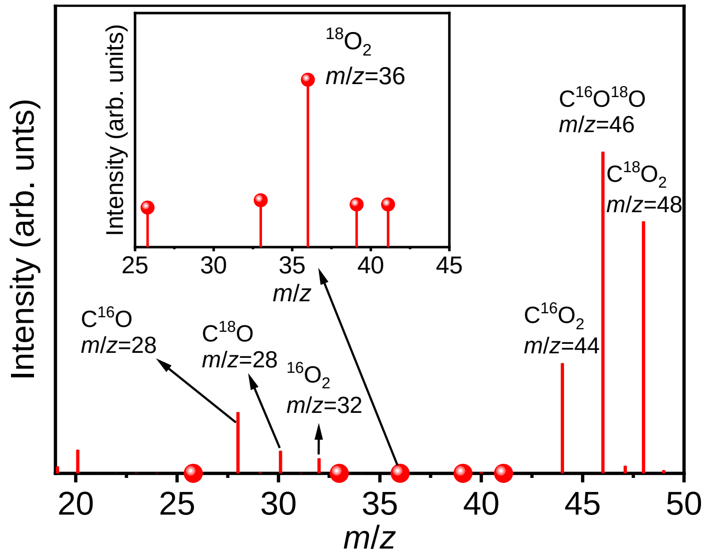

Supplementary Fig. 14 GC-MS spectrum of the products with $H_2{}^{18}O$. The inset shows an enlarged $^{18}O_2$ spectrum.

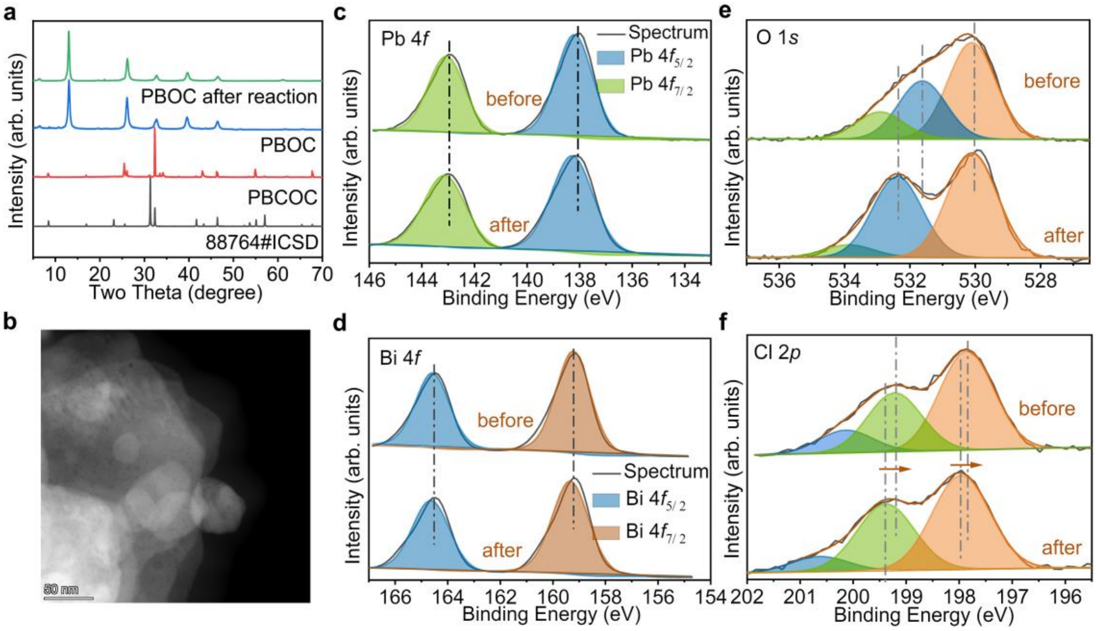

Supplementary Fig. 15 Characterization of the ultrathin PBOC before and after Photocatalytic CO_2RR. a XRD patterns. **b** HADDF-STEM image of ultrathin PBOC layers after photocatalytic CO_2RR. **c** Pb 4*f*. **d** Bi 4*f*. **e** O 1*s* and **f** Cl 2*p* XPS spectra.

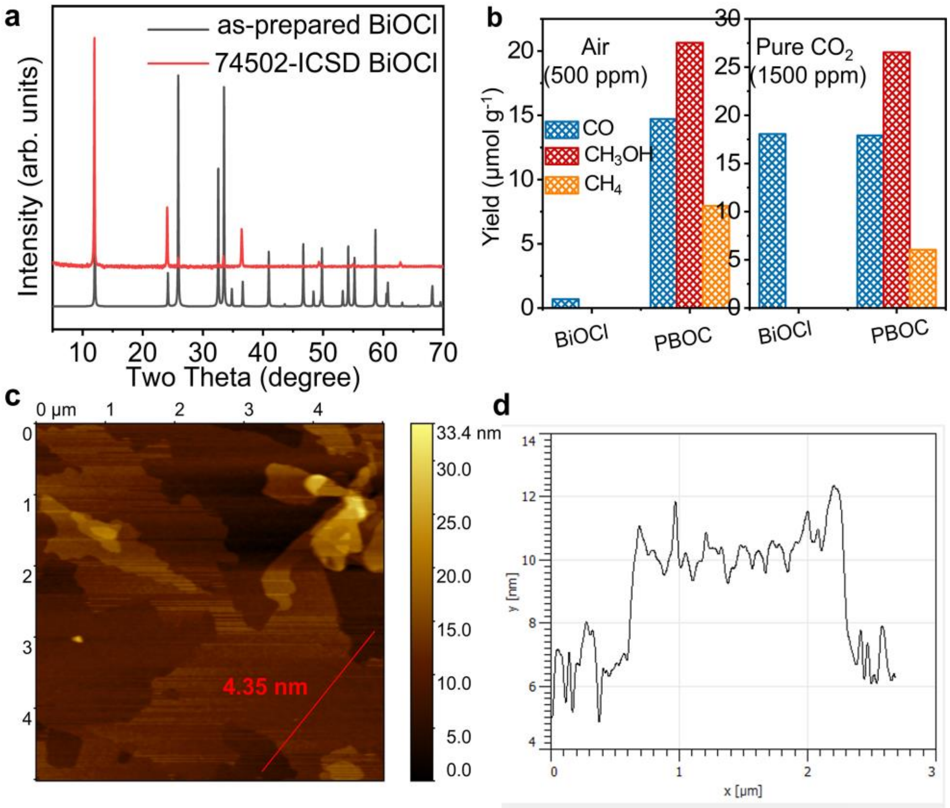

Supplementary Fig. 16 Characterization and photocatalytic CO_2RR performances of BiOCl nanosheets. a XRD pattern. **b** CO_2 reduction activities of BiOCl nanosheets and ultrathin PBOC layers under full-spectrum light irradiation. **c** AFM image and **d** the corresponding height profile. Error bars are means ± standard deviation of three replicates.

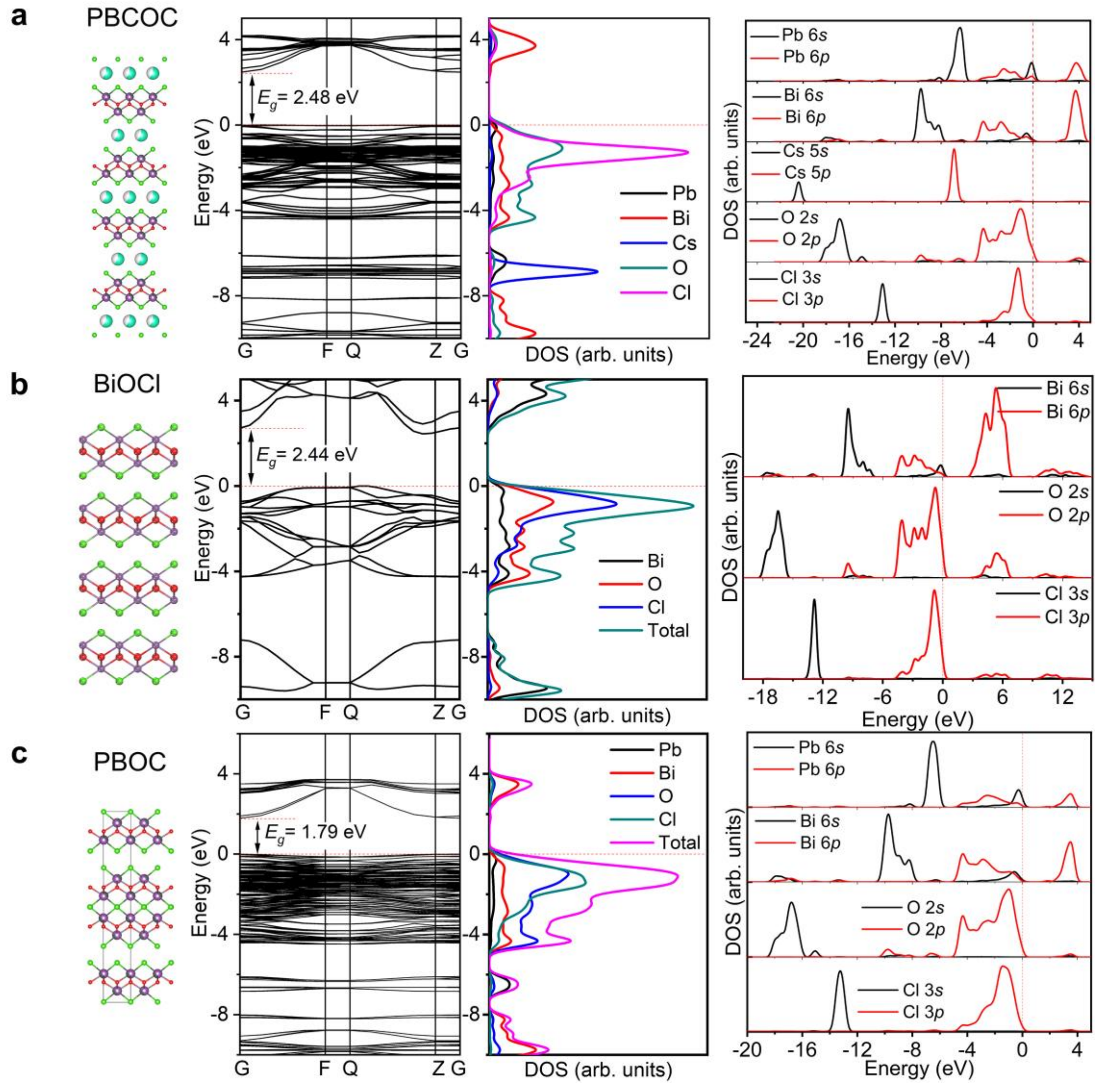

Supplementary Fig. 17 DFT calculation results. Crystal structures, band structures, electron density of states (DOS) and partial DOS plots from DFT calculation analysis: **a** bulk PBCOC. **b** BiOCl and **c** PBOC.

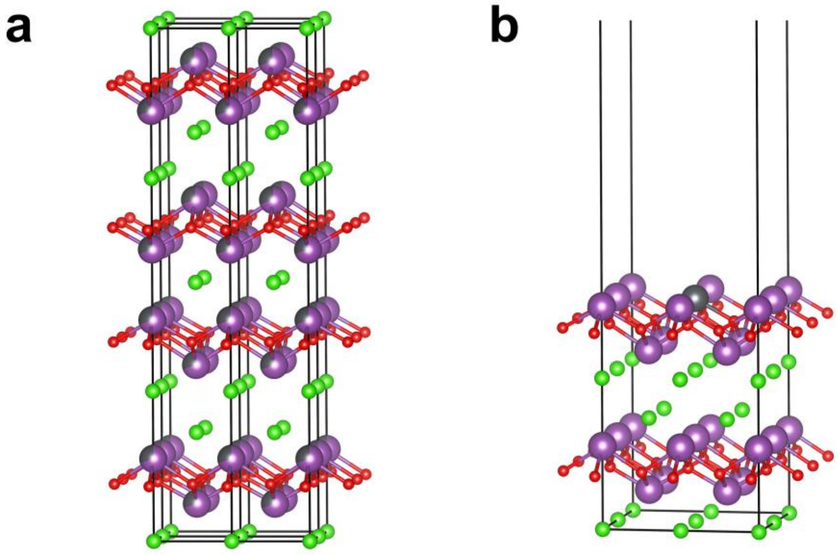

Supplementary Fig. 18 Bulk PBOC crystal (a) and PBOC surface model (b).

Supplementary Note 1

DFT calculation for evaluating the CO_2RR steps of ultrathin PBOC[1–9].

We employed DFT to explore the subsequent CO_2RR steps of ultrathin PBOC, from which three possible reaction pathways and reaction mechanisms of CO_2RR to CO, CH_3OH and CH_4 were considered, respectively. Detailed reaction pathways and related calculated free energy are shown as following[10–12]:

(1) Pathway I CO: CO_2 →*CO_2 (−0.651 eV) →*COOH (−0.381 eV) →*CO (−0.035 eV) →CO (0.227 eV);

(2) Pathway II CH_3OH: CO_2 →*CO_2 (−0.651 eV) →*COOH (−0.381 eV) →*CO (−0.035 eV) →*CHO (−0.615 eV) →*CH_2O (−0.581 eV) →*CH_3O (−1.307 eV) → *CH_3OH (−0.398 eV) → CH_3OH (0.086 eV);

(3) Pathway III CH_4: CO_2→*CO_2 (−0.651 eV) →*COOH (−0.381 eV) →*CO (−0.035 eV) →*CHO (−0.615 eV) →*CHOH (0.600 eV) →*CH (0.390 eV) → *CH_2 (−0.224 eV) → *CH_3 (−0.979 eV) → *CH_4 (−1.560 eV) → CH_4 (−1.113 eV).

Supplementary Table 4 Calculated zero-point energy (E_{ZPE}) and entropy correction ($T\Delta S$) for the molecules at T = 298.15 K[10,13].

Species	E_{ZPE} (eV)	$T\Delta S$ (eV)	E_{ZPE} (eV)*	$T\Delta S$ (eV)*
CO_2	0.311	0.467	0.31	0.65
H_2	0.272	0.421	0.27	0.41
H_2O	0.570	0.601	0.57	0.67
CO	0.130	0.611	0.14	0.67
CH_3OH	1.366	0.734	1.35	0.79
CH_4	1.192	0.640	1.2	0.6

Supplementary Table 5 Calculated zero-point energies (E_{ZPE}), entropy (S), heat capacity (C_p), enthalpy (H), free energy corrections (G_c) for adsorbates.

Species	E_{ZPE} (kcal mol^{-1})	S (kcal mol^{-1})	C_p (kcal mol^{-1})	H (kcal mol^{-1})	G_c (kcal mol^{-1})
*CO_2	60.819	467.205	315.928	124.745	-14.552
*COOH	68.153	477.29	323.689	133.736	-8.568
*CO	57.738	471.141	313.423	121.48	-18.99
*CHO	62.208	500.848	329.507	130.18	-19.148
*CH_2O	68.494	500.51	329.234	136.352	-12.875
*CH_3O	76.748	499.497	332.243	145.307	-3.618
*CH_3OH	82.668	497.975	327.49	150.594	2.123
*CHOH	68.525	504.894	332.74	137.261	-13.274
*CH	59.34	487.27	322.145	125.348	-19.931
*CH_2	66.548	485.747	321.12	132.026	-12.8
*CH_3	73.622	491.362	329.255	141.261	-5.238
*CH_4	79.156	511.667	328.753	147.542	-5.011

Supplementary Table 6 Summary of state-of-the-art photocatalytic CO_2RR catalysts.

Photocatalyst	Synthesis Method	Light Source	Experimental Condition	Main Product	Yield (μmol g^{-1} h^{-1})	Reference
Ultrathin $Pb_{0.6}Bi_{1.4}O_2Cl_2$	Desalinization	300 W xenon lamp	Air, gas-solid	CO	5.16	**Our work**
				CH_3OH	1.98	
				CH_4	0.62	
Ultrathin $Pb_{0.6}Bi_{1.4}O_2Cl_2$	Desalinization	300 W xenon lamp	1500 ppm, gas-solid	CO	4.48	**Our work**
				CH_3OH	6.64	
				CH_4	1.51	
Bulk $Pb_{0.6}Bi_{1.4}Cs_{0.6}O_2Cl_2$	Solid-state method	300 W xenon lamp	1500 ppm, gas-solid	CO	0.62	**Our work**
				CH_3OH	0.91	
				CH_4	1.20	
Single Cu Atom/Crystalline g-C_3N_4	Pyrolysis	300 W xenon lamp	Pure CO_2 gas, gas-solid	CO<br>CH_4	3.086<br>trace	14

Oxygen-vacancy $Bi_4Ti_3O_{12}$ ultrathin nanosheets	A combined hydrothermal and post-reduction process.	300 W xenon lamp	Pure CO_2 gas, gas-solid	CO	11.7	15
Atomically-thin Bi_2MoO_6 nanosheets with vacancy pairs	A template-directed strategy	300 W xenon lamp	Pure CO_2 gas, gas-solid	CO	3.62	16
BiOCl nanoplates with photoinduced oxygen vacancies	Solvothermal method and 500 W xenon lamp irradiation	500 W xenon lamp	Pure CO_2 gas, liquid-solid	CO	1.01	17
				CH_4	0.15	
ZnAl-LDH nanosheets	a reverse micelle method	300 W xenon lamp	Pure CO_2 gas, gas-solid	CO	7.6	18
$TiO_2/CsPbBr_3$	Electrostatic mixing	300 W xenon lamp	Pure CO_2 gas, aqueous-solid with sacrificial agent	CO	9.02	19
Cs_xWO_3	Solvothermal method	300 W xenon lamp	Air, gas-solid	CO	0.07	20
				CH_4	0.03	

				CH_3OH<br>HCHO	4.26<br>1.63	
Atomically thin Bi_2WO_6	Solvothermal method	300 W xenon lamp	Pure CO_2 gas	CO	7.12	21
				CH_4	0.63	
Ultrathin BiOBr	Solvothermal method	300 W xenon lamp (λ > 400 nm)	Pure CO_2 gas, gas-solid	CO	2.67	22
				CH_4	0.16	
Perovskite-like $PbBiO_2Br$	Solvothermal method	300 W xenon lamp	Pure CO_2 gas, gas-solid	CO	1.24	23
Bismuth-rich $Bi_4O_5Br_2$ hollow microspheres	Solvothermal method	300 W xenon lamp	Pure CO_2 gas, gas-solid, water	CO	3.16	24
				CH_4	0.5	
$Cs_3Bi_2I_9$ perovskite nanocrystals	Ultrasonication top-down method	32 W UV lamp	Pure CO_2 gas, gas-solid, water	CO	7.76	25
				CH_3OH	1.49	
Monolayer N-doped graphene on CdS hollow spheres	Chemical vapor deposition	A 350 W Xenon lamp (λ > 420 nm)	Pure CO_2 gas, gas-solid, water	CO	2.59	26
				CH_4	0.33	

Cs_2SnI_6 Perovskite Nanocrystal/SnS_2 Nanosheet Heterojunction	Solvothermal method	A 150 mW cm^{-2} Xenon lamp (λ > 400 nm)	Pure CO_2 gas, gas-solid, water, CH_3OH	CH_4	3.03	27
Ultrathin MXene/Bi_2WO_6 Nanosheets	Etching and solvothermal method	300 W xenon lamp	Pure CO_2 gas, gas-solid, water	CH_4	1.78	28
				CH_3OH	0.44	
UiO-66/carbon nitride nanosheet heterogeneous photocatalyst	Solvothermal method	A 300 W xenon lamp (800 nm >λ > 400 nm)	Pure CO_2 gas, gas-solid, water	CO	9.9	29
$Bi_2O_2(OH)(NO_3)$ with surface Br ions	Solvothermal method	300 W xenon lamp	Pure CO_2 gas, gas-solid, water	CO	8.12	30
Cu Single Atoms Decorated on UiO-66-NH_2	Oil bath method and photoinduction method	A 300 W xenon lamp (λ > 400 nm)	Pure CO_2 gas, gas-solid, water	CH_3OH	5.33	31
				C_2H_5OH	4.22	
Atomically thin $CuIn_5S_8$ layers	Solvothermal method	A 300 W xenon lamp (λ > 420 nm)	Pure CO_2 gas, gas-solid, water	CH_4	8.7	32

Supplementary References

1. Delley, B. An all‐electron numerical method for solving the local density functional for polyatomic molecules. *J. Chem. Phys.* **92**, 508–517 (1990).

2. Delley, B. From molecules to solids with the DMol^3 approach. *J. Chem. Phys.* **113**, 7756–7764 (2000).

3. Perdew, J. P., Burke, K. & Ernzerhof, M. Generalized gradient approximation made simple. *Phys. Rev. Lett.* **77**, 3865–3868 (1996).

4. Hammer, B., Hansen, L. B. & Nørskov, J. K. Improved adsorption energetics within density-functional theory using revised Perdew-Burke-Ernzerhof functionals. *Phys. Rev. B* **59**, 7413–7421 (1999).

5. Du, A. *et al.* Hybrid Graphene and Graphitic Carbon Nitride Nanocomposite: Gap Opening, Electron–Hole Puddle, Interfacial Charge Transfer, and Enhanced Visible Light Response. *J. Am. Chem. Soc.* **134**, 4393–4397 (2012).

6. Delley, B. Hardness conserving semilocal pseudopotentials. *Phys. Rev. B* **66**, 155125 (2002).

7. Liu, P. & Rodriguez, J. A. Catalysts for hydrogen evolution from the [NiFe] hydrogenase to the Ni 2P(001) surface: The importance of ensemble effect. *J. Am. Chem. Soc.* **127**, 14871–14878 (2005).

8. Nørskov, J. K. *et al.* Origin of the Overpotential for Oxygen Reduction at a Fuel-Cell Cathode. *J. Phys. Chem. B* **108**, 17886–17892 (2004).

9. Rossmeisl, J., Logadottir, A. & Nørskov, J. K. Electrolysis of water on (oxidized) metal surfaces. *Chem. Phys.* **319**, 178–184 (2005).

10. Hansen, H. A., Varley, J. B., Peterson, A. A. & Nørskov, J. K. Understanding Trends in the Electrocatalytic Activity of Metals and Enzymes for CO_2 Reduction to CO. *J. Phys. Chem. Lett.* **4**, 388–392 (2013).

11. Chai, G. L. & Guo, Z. X. Highly effective sites and selectivity of nitrogen-doped graphene/CNT catalysts for CO_2 electrochemical reduction. *Chem. Sci.* **7**, 1268–1275 (2016).

12. Li, Y., Li, B., Zhang, D., Cheng, L. & Xiang, Q. Crystalline Carbon Nitride Supported Copper Single Atoms for Photocatalytic CO_2 Reduction with Nearly 100% CO Selectivity. *ACS Nano* **14**, 10552–10561 (2020).

13. Peterson, A. A., Abild-Pedersen, F., Studt, F., Rossmeisl, J. & Nørskov, J. K. How copper catalyzes the electroreduction of carbon dioxide into hydrocarbon fuels. *Energy Environ. Sci.* **3**, 1311–1315 (2010).

14. Wu, J. *et al.* Efficient Visible-Light-Driven CO_2 Reduction Mediated by Defect-Engineered BiOBr Atomic Layers. *Angew. Chem. Int. Ed.* **57**, 8719–8723

(2018).

15. Liu, L. *et al.* Cooperation of oxygen vacancies and 2D ultrathin structure promoting CO_2 photoreduction performance of $Bi_4Ti_3O_{12}$. *Sci. Bull.* **65**, 934–943 (2020).

16. Di, J. *et al.* Atomically-thin Bi_2MoO_6 nanosheets with vacancy pairs for improved photocatalytic CO_2 reduction. *Nano Energy* **61**, 54–59 (2019).

17. Zhang, L., Wang, W., Jiang, D., Gao, E. & Sun, S. Photoreduction of CO_2 on BiOCl nanoplates with the assistance of photoinduced oxygen vacancies. *Nano Res.* **8**, 821–831 (2015).

18. Zhao, Y. *et al.* Defect-Rich Ultrathin ZnAl-Layered Double Hydroxide Nanosheets for Efficient Photoreduction of CO_2 to CO with Water. *Adv. Mater.* **27**, 7824–7831 (2015).

19. Xu, F. *et al.* Unique S-scheme heterojunctions in self-assembled TiO_2/$CsPbBr_3$ hybrids for CO_2 photoreduction. *Nat. Commun.* **11**, 1–9 (2020).

20. Wu, X. *et al.* Photocatalytic CO_2 Conversion of $M_{0.33}WO_3$ Directly from the Air with High Selectivity: Insight into Full Spectrum-Induced Reaction Mechanism. *J. Am. Chem. Soc.* **141**, 5267–5274 (2019).

21. Liu, Y., Shen, D., Zhang, Q., Lin, Y. & Peng, F. Enhanced photocatalytic CO_2 reduction in H_2O vapor by atomically thin Bi_2WO_6 nanosheets with hydrophobic and nonpolar surface. *Appl. Catal. B* **283**, 119630 (2021).

22. Ye, L. *et al.* Thickness-ultrathin and bismuth-rich strategies for BiOBr to enhance photoreduction of CO_2 into solar fuels. *Appl. Catal. B* **187**, 281–290 (2016).

23. Wang, B. *et al.* Sacrificing ionic liquid-assisted anchoring of carbonized polymer dots on perovskite-like $PbBiO_2Br$ for robust CO_2 photoreduction. *Appl. Catal. B* **254**, 551–559 (2019).

24. Jin, X. *et al.* A bismuth rich hollow $Bi_4O_5Br_2$ photocatalyst enables dramatic CO_2 reduction activity. *Nano Energy* **64**, 103955 (2019).

25. Bhosale, S. S. *et al.* Mechanism of Photocatalytic CO_2 Reduction by Bismuth-Based Perovskite Nanocrystals at the Gas–Solid Interface. *J. Am. Chem. Soc.* **141**, 20434–20442 (2019).

26. Bie, C., Zhu, B., Xu, F., Zhang, L. & Yu, J. In Situ Grown Monolayer N-Doped Graphene on CdS Hollow Spheres with Seamless Contact for Photocatalytic CO_2 Reduction. *Adv. Mater.* **31**, (2019).

27. Wang, X. D. *et al.* In Situ Construction of a Cs_2SnI_6 Perovskite Nanocrystal/SnS_2 Nanosheet Heterojunction with Boosted Interfacial Charge Transfer. *J. Am. Chem. Soc.* **141**, 13434–13441 (2019).

28. Cao, S., Shen, B., Tong, T., Fu, J. & Yu, J. 2D/2D Heterojunction of Ultrathin MXene/Bi_2WO_6 Nanosheets for Improved Photocatalytic CO_2 Reduction. *Adv. Funct. Mater.* **28**, 1–11 (2018).

29. Shi, L., Wang, T., Zhang, H., Chang, K. & Ye, J. Electrostatic Self-Assembly of Nanosized Carbon Nitride Nanosheet onto a Zirconium Metal-Organic Framework for Enhanced Photocatalytic CO_2 Reduction. *Adv. Funct. Mater.* **25**, 5360–5367 (2015).

30. Hao, L. *et al.* Surface-Halogenation-Induced Atomic-Site Activation and Local Charge Separation for Superb CO_2 Photoreduction. *Adv. Mater.* **31**, 1–7 (2019).

31. Wang, G. *et al.* Photoinduction of Cu Single Atoms Decorated on UiO-66-NH_2 for Enhanced Photocatalytic Reduction of CO_2 to Liquid Fuels. *J. Am. Chem. Soc.* **142**, 19339–19345 (2020).

32. Li, X. *et al.* Selective visible-light-driven photocatalytic CO_2 reduction to CH_4 mediated by atomically thin $CuIn_5S_8$ layers. *Nat. Energy* **4**, 690–699 (2019).
